# Chikungunya in a co-circulation setting: diagnostic discrepancies and clinical variation between RT-PCR-positive cases and notified data in Foz do Iguaçu, Brazil

**DOI:** 10.1590/1414-431X2026e15563

**Published:** 2026-08-21

**Authors:** W.B. Costa, B.B. de Pinho, L.M.M. Crisóstomo, E. Werncke, L.F.B. Zarpelon, M.C. Gross, R. Zazula, M.L. Terencio

**Affiliations:** 1 Laboratório de Pesquisa em Ciências Médicas, Instituto Latino-Americano de Ciências da Vida e da Natureza, Universidade Federal de Integração Latino-Americana, Foz do Iguaçu, PR, Brasil

**Keywords:** Chikungunya virus, Arboviral disease, Diagnosis, Health surveillance

## Abstract

Chikungunya virus infection remains a diagnostic challenge in regions with co-circulation of dengue, where overlapping clinical manifestations frequently lead to misclassification. This study compared the clinical and epidemiological profiles of chikungunya cases confirmed by molecular diagnosis (RT-PCR) with cases notified to the Brazilian National Notifiable Diseases Information System (SINAN) during the 2023 epidemic in Foz do Iguaçu, southern Brazil, aiming to identify diagnostic discrepancies, symptom patterns, and potential selection biases. A retrospective analytical study was conducted using two datasets: 201 RT-PCR confirmed cases obtained from medical records and 1,964 suspected cases reported to SINAN. Descriptive analyses, bivariate tests, hierarchical cluster analysis, and multiple correspondence analysis were performed. Among RT-PCR confirmed cases, the initial clinical diagnostic error rate was 58.7%. Arthralgia was the primary predictor of a correct clinical diagnosis (OR=3.08) and was more prevalent among RT-PCR confirmed cases than among laboratory-confirmed cases reported to SINAN. In contrast, arthritis and retro-orbital pain were more frequent among cases confirmed by clinical-epidemiological criteria in SINAN than among RT-PCR confirmed cases. Symptom clustering revealed distinct clinical profiles, with an articular pattern accounting for the highest proportion of RT-PCR-positive cases. Hospitalization rates varied by confirmation criteria, suggesting a selection bias toward laboratory testing. These findings demonstrated discrepancies between molecularly confirmed cases and routine surveillance data, as well as a high rate of initial clinical misdiagnosis. The results highlight the need to refine clinical-epidemiological criteria, prioritize arthralgia as a key diagnostic symptom, and account for chikungunya's clinical heterogeneity to improve diagnosis and epidemiological surveillance.

## Introduction

Chikungunya fever, caused by the Chikungunya virus (CHIKV), an alphavirus of the family Togaviridae, constitutes a significant public health concern in tropical and subtropical regions (1,2). Transmission occurs primarily through *Aedes aegypti* and *Aedes albopictus* mosquitoes, which also serve as vectors for dengue and Zika viruses, facilitating the concurrent circulation of these arboviruses and recurrent epidemics (3,4). Following its global reemergence in 2004 and subsequent introduction into the Americas in 2013, CHIKV has disseminated rapidly, with Brazil reporting a substantial number of cases and emerging as one of the most affected countries in the region (4,5).

The acute phase of chikungunya is characterized by sudden-onset fever, severe myalgia, and polyarthralgia, frequently affecting multiple joints in a symmetrical distribution (6,7). Additional common symptoms include headache, fatigue, and maculopapular rash (8). While the acute illness is generally self-limiting, a considerable proportion of patients experience chronic manifestations, particularly persistent arthralgia or inflammatory arthritis, which can persist for months or years and substantially reduce quality of life (6,9,10). Furthermore, atypical and severe presentations involving neurological, cardiac, or systemic complications have been documented, particularly among older adults and individuals with preexisting comorbidities (11-13).

Accurate diagnosis of chikungunya is challenging, especially in regions where other arboviruses with similar clinical presentations, such as dengue and Zika, are endemic (14,15). Laboratory confirmation using reverse transcription polymerase chain reaction (RT-PCR) during the viremic phase or serological assays in later stages is regarded as the reference standard (16,17). Nevertheless, limited access to laboratory testing, particularly during large outbreaks, often necessitates reliance on clinical and epidemiological criteria for case definition and notification (3,7).

In Brazil, suspected chikungunya cases are reported to the National Notifiable Diseases Information System (*Sistema de Informação de Agravos de Notificação*, SINAN), which utilizes both laboratory and clinical-epidemiological confirmation criteria. The ability of these criteria to differentiate chikungunya from other acute febrile illnesses, particularly dengue, remains uncertain and may result in diagnostic misclassification and biased surveillance data (15,18). Robust epidemiological surveillance is critical for monitoring viral transmission, assessing disease burden, and informing public health interventions (3,19).

Foz do Iguaçu, situated in a tri-border area in southern Brazil, has experienced recurrent arbovirus epidemics, with documented co-circulation of dengue, Zika, and chikungunya viruses. In this setting, discrepancies between cases confirmed by highly specific molecular methods and those recorded in routine surveillance systems may obscure the actual clinical and epidemiological profile of chikungunya.

This study aimed to compare the demographic, clinical, and outcome characteristics of chikungunya cases confirmed by RT-PCR with those of cases reported to SINAN in Foz do Iguaçu during the 2023 epidemic. Additionally, the accuracy of initial clinical diagnoses among RT-PCR-confirmed cases and factors associated with diagnostic discrepancies were assessed to identify potential biases and generate evidence to enhance clinical practice and epidemiological surveillance.

## Material and Methods

### Study design and data sources

An observational, analytical, and comparative study was conducted using two independent datasets of chikungunya cases recorded in Foz do Iguaçu, Paraná, Brazil, primarily during the 2023 epidemic period. This study was approved by the Human Research Ethics Committee (CAAE No. 84396024.2.0000.8527).

The first dataset, designated as the “RT-PCR-positive dataset”, included 201 cases confirmed for chikungunya by molecular diagnosis using RT-PCR, the reference standard for acute infection. Data were manually extracted from medical records of patients who sought care at local health services during the epidemic period. This dataset comprised 25 variables encompassing demographic characteristics (age and sex), clinical features (presence or absence of 12 predefined symptoms coded as binary variables, date of symptom onset, date of laboratory confirmation, initial clinical diagnostic hypothesis recorded by the attending physician, and tourniquet test result), and clinical outcomes (discharge, hospital admission, observation in an emergency care unit, or death).

The second dataset, termed the “SINAN/DATASUS dataset”, comprised 1,964 records of suspected chikungunya cases reported to SINAN in 2023. Data were systematically extracted from the official municipal epidemiological surveillance database. This dataset included 136 variables, such as demographic information, clinical manifestations (coded as “yes” or “no”), diagnostic confirmation criteria (laboratory, clinical-epidemiological, or under investigation), laboratory test results (when available, including serology and RT-PCR), reported comorbidities, and clinical outcomes (recovery, death due to chikungunya, death from other causes, unknown, or not reported).

### Data processing and harmonization

Both datasets were subjected to consistency checks and quality control procedures. In the RT-PCR positive dataset, no substantial missing data were identified for the main variables, including the initial clinical diagnostic hypothesis. In the SINAN/DATASUS dataset, data completeness was evaluated, and variables were categorized by the proportion of missing values, distinguishing between high (>20%) and low (<5%) levels of missingness.

To facilitate comparative analyses, variables were harmonized across datasets. Categorical variables, such as sex, were standardized. Clinical symptoms were converted to a binary format. Age was analyzed as a continuous variable in both datasets, and age groups were created for stratified analyses. Outcome variables were recoded to ensure comparability between data sources.

### Statistical analysis

Statistical analyses were conducted using R software version 4.3.3 (20) with the following packages: stats (20), cluster (21), FactoMineR (22), and fmsb (23). Additional analyses were performed in JavaScript. All raw data and analytical scripts are publicly available in a GitHub repository (https://github.com/WelCode99/ArbovirosesDATA.git).

Descriptive analyses involved calculating absolute and relative frequencies with 95% confidence intervals (95%CI) for categorical variables. For continuous variables, measures of central tendency (mean and median) and dispersion (standard deviation, interquartile range, and range) were computed. The normality of age distribution was assessed using the Shapiro-Wilk (24) and Kolmogorov-Smirnov (25) tests, as well as skewness and kurtosis analyses.

In the RT-PCR-positive dataset, the accuracy of the initial clinical diagnosis was assessed by calculating the proportion of cases in which the recorded diagnostic hypothesis included “chikungunya” rather than alternative diagnoses. Odds ratios (OR) and 95%CI were estimated to evaluate associations between the presence or absence of symptoms, tourniquet test results, and diagnostic accuracy. The mean number of reported symptoms was compared between correctly and incorrectly diagnosed cases.

Comparative analyses between RT-PCR confirmed cases and SINAN/DATASUS subgroups were conducted using Pearson's chi-squared test or Fisher's exact test (26) for categorical variables, as appropriate. Differences in mean age were evaluated using Student's *t*-test following verification of variance homogeneity with Levene's test (27). Crude OR and 95%CI were calculated to compare symptom prevalence between groups.

Hospitalization rates and indicators of disease severity were compared using chi-squared tests. A multivariable logistic regression model was developed to identify factors independently associated with hospitalization, adjusting for potential confounders, such as age, sex, diagnostic group, and key clinical manifestations. Model performance was assessed using the area under the receiver operating characteristic curve (AUC-ROC) (28) and the Hosmer-Lemeshow goodness-of-fit test (29).

To identify natural symptom profiles among RT-PCR-confirmed cases, hierarchical cluster analysis was conducted using Jaccard distance (30) and the Ward D2 linkage method (31). The optimal number of clusters was determined using silhouette width (32) and the Calinski-Harabasz index (33).

Multiple correspondence analysis (MCA) (34) was applied to clinical and demographic variables, as well as PCR status (in the SINAN dataset), to explore associations and visualize symptom patterns in a multidimensional space.

To assess selection bias for molecular testing, a logistic regression model was constructed using the SINAN/DATASUS dataset to estimate the probability of undergoing RT-PCR testing based on demographic and clinical characteristics. Inverse probability weighting (IPW), derived from propensity-score testing, was applied to adjust symptom prevalence estimates and mitigate selection bias.

A two-sided significance level of P<0.05 was adopted for all statistical analyses.

## Results

### Sample characterization and demographic comparison

The RT-PCR-positive dataset comprised 201 laboratory-confirmed chikungunya cases, with a mean age of 41.2 years (SD=18.9), median age of 41 years (IQR 26-58), and a higher proportion of female individuals (60.7%). The most represented age groups were 20-29 years (20.9%) and 50-59 years (17.4%).

The SINAN/DATASUS dataset included 1,964 notified records, of which 1,101 (56.1%) were classified as confirmed cases (558 by laboratory criteria and 543 by clinical-epidemiological criteria), 851 (43.3%) as discarded cases, and 12 (0.6%) without classification.

After harmonization of variables, no statistically significant differences were observed in sex distribution or mean age between the SINAN/DATASUS subgroups and the RT-PCR confirmed group (P>0.05 for all comparisons). Age group distributions were also similar across groups, with a higher concentration of adults aged 20-59 years.

### Accuracy of the initial clinical diagnosis and associated factors (RT-PCR-positive group)

Among the 201 RT-PCR confirmed cases, the accuracy of the initial clinical diagnosis was 41.29%, whereas 58.71% of patients received an initial diagnostic hypothesis other than chikungunya.

**Figure 1:**
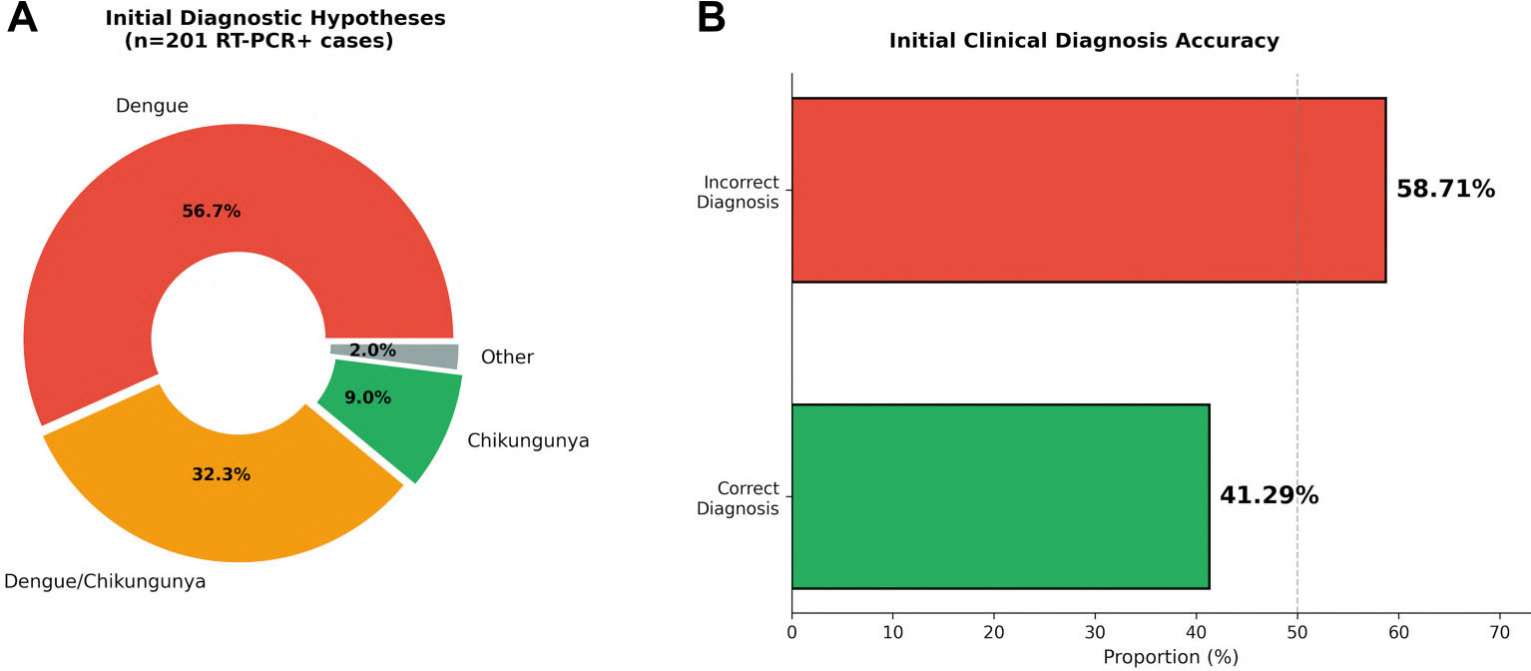
Initial diagnostic hypotheses and clinical accuracy in RT-PCR-confirmed cases.

The distribution of initial diagnostic hypotheses is shown in Figure 1. Dengue was recorded as the sole initial diagnosis in 114 cases (56.7%), dengue or chikungunya as differential diagnoses in 65 cases (32.3%), chikungunya alone in 18 cases (9.0%), and other conditions in 4 cases (2.0%).

**Figure 2:**
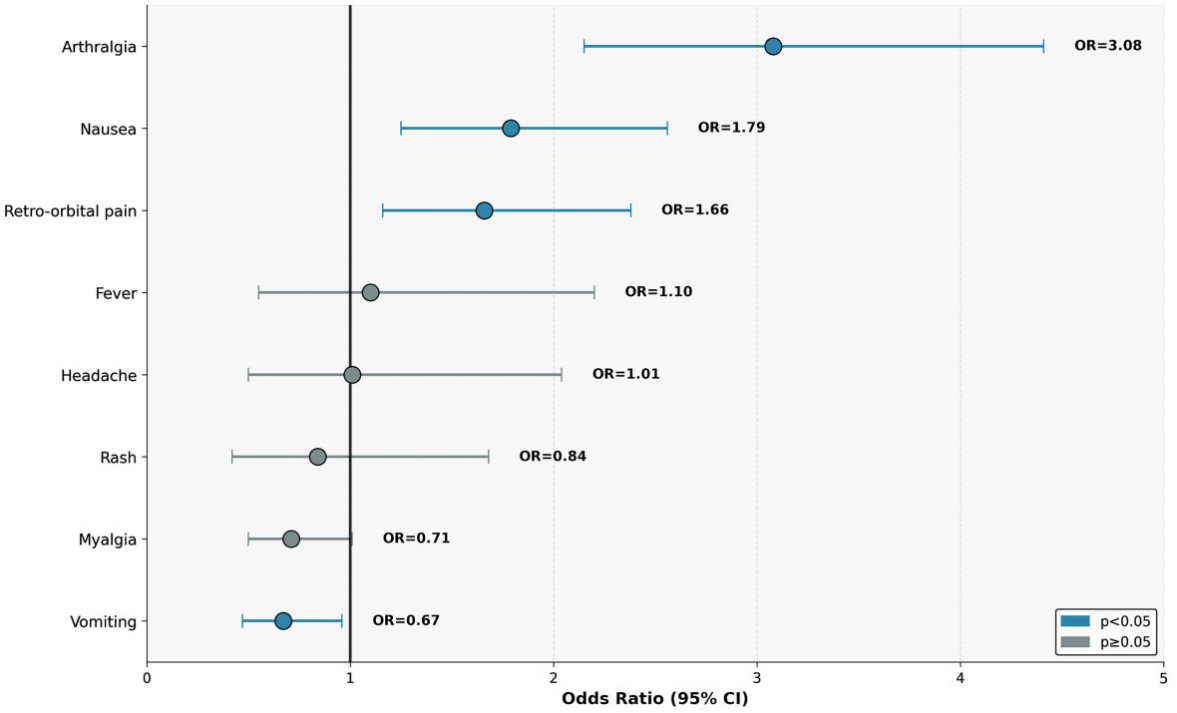
Factors associated with initial clinical diagnosis accuracy (RT-PCR+ cases, n=201). Statistical analysis: Pearson's chi-squared test or Fisher's exact test, as appropriate; odds ratios (OR) with 95% confidence intervals.

The probability of a correct initial clinical diagnosis (including chikungunya) was significantly influenced by the presence of specific symptoms and the tourniquet test (Table 1 and Figure 2).

**Table 1:** Diagnostic accuracy associated with clinical symptoms.

Associated factor	Diagnostic accuracy with symptom (%)	Diagnostic accuracy without symptom (%)	Difference (p.p.)	Odds Ratio (OR)
Arthralgia	49.63	24.24	+25.39	3.08
Nausea	52.00	37.75	+14.25	1.79
Retro-orbital pain	52.63	40.11	+12.52	1.66
Fever	41.67	39.39	+2.27	1.10
Headache	41.41	41.10	+0.31	1.01
Rash	37.50	41.81	-4.31	0.84
Myalgia	39.49	47.73	-8.24	0.71
Vomiting	33.33	42.69	-9.36	0.67

Statistical analysis: Pearson's chi-squared test or Fisher's exact test, as appropriate.

Arthralgia was significantly associated with a higher likelihood of a correct initial diagnosis (OR=3.08), as were nausea and retro-orbital pain. In contrast, myalgia (OR=0.71) and vomiting (OR=0.67) were associated with reduced diagnostic accuracy.

A positive tourniquet test was associated with lower diagnostic accuracy (21.43%) compared with negative test results (44.08%). The mean number of reported symptoms was slightly higher among correctly diagnosed cases (3.70) than among incorrectly classified cases (3.42).

### Comparison of clinical and symptomatic profiles (RT-PCR-positive *vs* SINAN/DATASUS)

**Figure 3:**
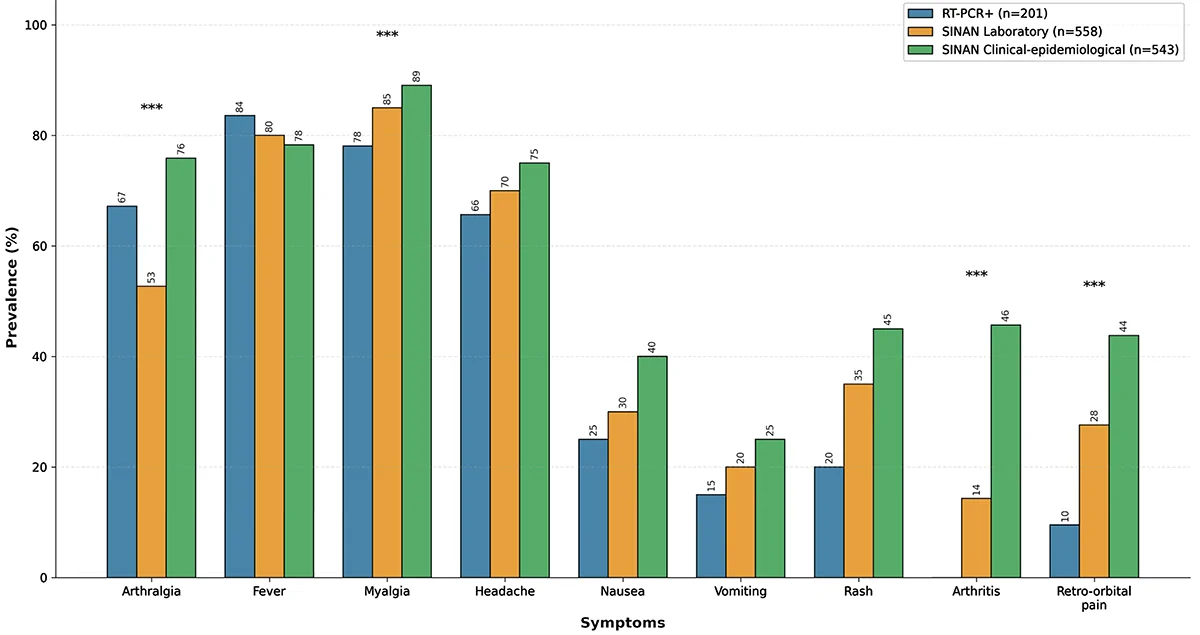
Comparison of symptom prevalence between diagnostic confirmation methods. ***P<0.001; Pearson's chi-squared test or Fisher's exact test, as appropriate; crude odds ratios with 95% confidence intervals. SINAN: Brazilian National Notifiable Diseases Information System.

Comparison of symptom frequencies between RT-PCR confirmed cases and SINAN/DATASUS subgroups revealed marked clinical heterogeneity (Figure 3).

Arthralgia was more prevalent in the RT-PCR-positive group (67.2%) than among laboratory-confirmed cases in SINAN/DATASUS (52.7%; OR=1.84; 95%CI: 1.31-2.58; P<0.001), but less frequent than among cases confirmed by clinical-epidemiological criteria (75.9%; OR=0.65; 95%CI: 0.46-0.93; P=0.019).

Fever showed a similar prevalence in RT-PCR-positive cases (83.6%) and clinically confirmed cases (78.3%; P=0.113). In contrast, myalgia was less common in the RT-PCR-positive group (78.1%) than in clinically confirmed cases (89.1%; OR=0.44; 95%CI: 0.28-0.67; P<0.001).

Arthritis and retro-orbital pain exhibited distinct patterns. Arthritis was not reported among RT-PCR-positive cases (0%) but was recorded in 14.3% of laboratory-confirmed cases (P<0.001) and in 45.7% of clinically confirmed cases (P<0.001). Retro-orbital pain was also less frequent in the RT-PCR-positive group (9.5%) compared with laboratory-confirmed (27.6%; OR=0.27; 95%CI: 0.17-0.46; P<0.001) and clinically confirmed cases (43.8%; OR=0.13; 95%CI: 0.08-0.22; P<0.001) in the SINAN/DATASUS dataset.

Additional differences were observed for rash, nausea, and vomiting, further highlighting the divergence between the symptomatic profiles of RT-PCR-confirmed cases and those reported in the different SINAN/DATASUS subgroups.

### Hospitalization and severity analysis

**Figure 4:**
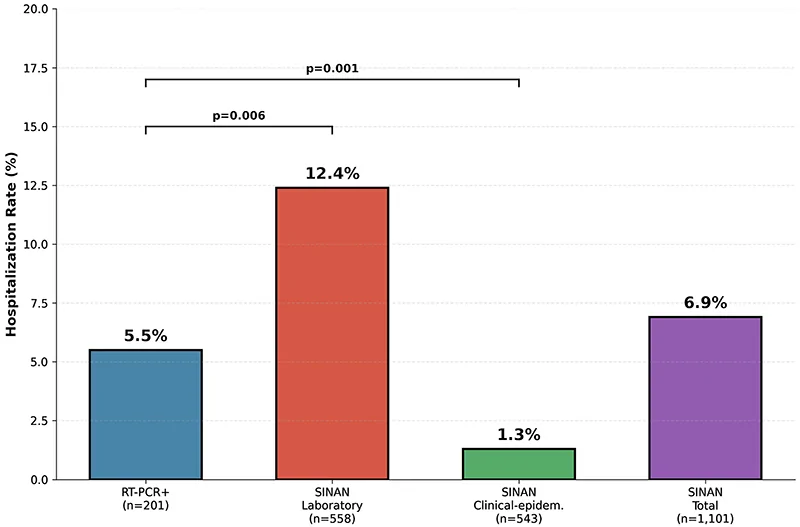
Hospitalization rates by diagnostic confirmation criteria. Statistical analysis: Pearson's chi-squared test. SINAN: Brazilian National Notifiable Diseases Information System.

Hospitalization rates differed significantly across the evaluated groups (Figure 4). Cases confirmed by laboratory criteria in the SINAN/DATASUS dataset had the highest hospitalization rate (12.4%), which was significantly higher than that observed in the RT-PCR-positive group (5.5%; P=0.006).

In contrast, cases confirmed by clinical-epidemiological criteria in the SINAN/DATASUS dataset had the lowest hospitalization rate (1.3%), which was significantly lower than that observed in the RT-PCR-positive group (P=0.001).

The overall hospitalization rate among confirmed cases in the SINAN/DATASUS dataset (6.9%) did not differ significantly from that observed in the RT-PCR positive group (P=0.469). Only 1 death (0.5%) was recorded in the RT-PCR-positive group.

**Figure 5:**
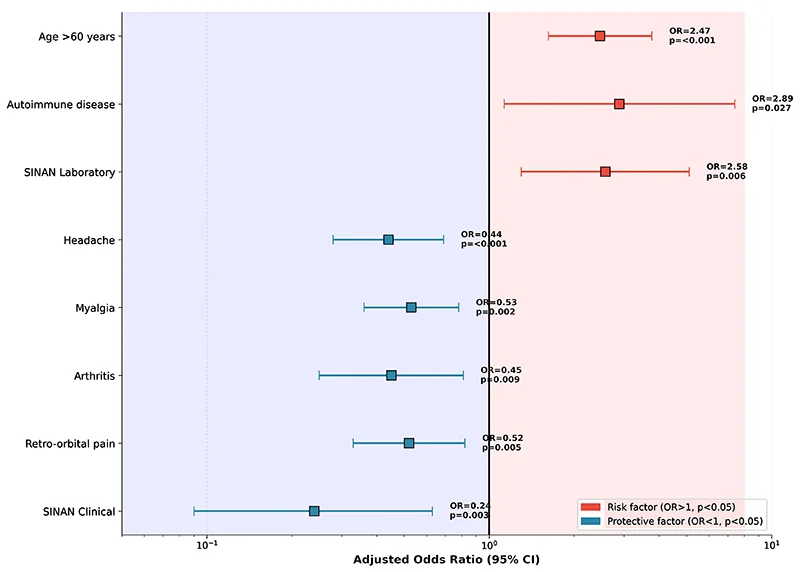
Factors associated with hospitalization. Statistical analysis: multivariable logistic regression; adjusted odds ratios (OR) with 95% confidence intervals (CI).

In the multivariable logistic regression model applied to the RT-PCR positive dataset (Figure 5), age older than 60 years (adjusted OR=2.47; 95%CI: 1.62-3.77; P<0.001) and the presence of autoimmune disease (adjusted OR=2.89; 95%CI: 1.13-7.41; P=0.027) were identified as independent risk factors for hospitalization. In contrast, headache (adjusted OR=0.44; P<0.001), myalgia (adjusted OR=0.53; P=0.002), arthritis (adjusted OR=0.45; P=0.009), and retro-orbital pain (adjusted OR=0.52; P=0.005) were associated with a lower probability of hospitalization.

In addition, laboratory confirmation in the SINAN/DATASUS dataset, compared with the RT-PCR-positive group, was associated with a higher likelihood of hospitalization (adjusted OR=2.58; P=0.006), whereas clinical-epidemiological confirmation was associated with a reduced probability of hospitalization (adjusted OR=0.24; P=0.003).

### Symptom patterns and selection bias

**Figure 6:**
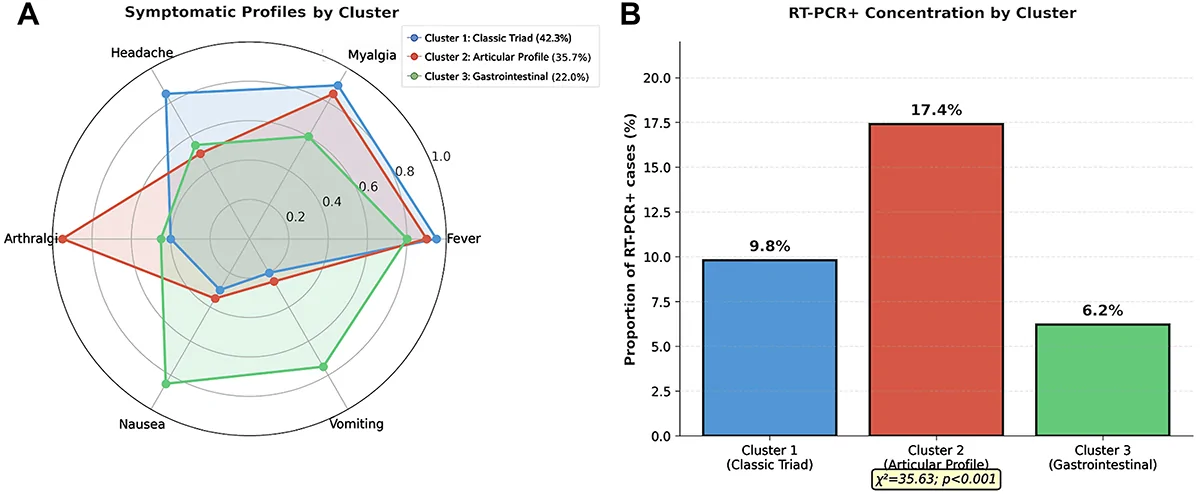
Cluster analysis: symptomatic profiles and RT-PCR+ case distribution. **A**, Symptomatic profiles by cluster. **B**, RT-PCR+ concentration by cluster. Statistical analysis: hierarchical cluster analysis using Ja ccard distance and Ward D2 linkage; Pearson's chi-squared test for comparison across clusters.

Cluster analysis performed on the RT-PCR-positive dataset identified three main clinical profiles (Figure 6). Cluster 1 was characterized by fever, myalgia, and headache (classical triad; 42.3%). Cluster 2 was marked by the combination of fever, myalgia, and arthralgia (articular profile; 35.7%). Cluster 3 comprised cases with fever, nausea, and vomiting (gastrointestinal profile; 22.0%). RT-PCR positive cases were significantly concentrated in cluster 2 (17.4% of cases belonging to this cluster) compared with cluster 1 (9.8%) and cluster 3 (6.2%) (χ^2^=35.63; P<0.001).

MCA confirmed the distinction between predominantly articular and gastrointestinal symptom profiles. Arthralgia, a central feature of cluster 2, also emerged as the main predictor of correct initial clinical diagnosis, reinforcing the relevance of this symptomatic profile.

**Figure 7:**
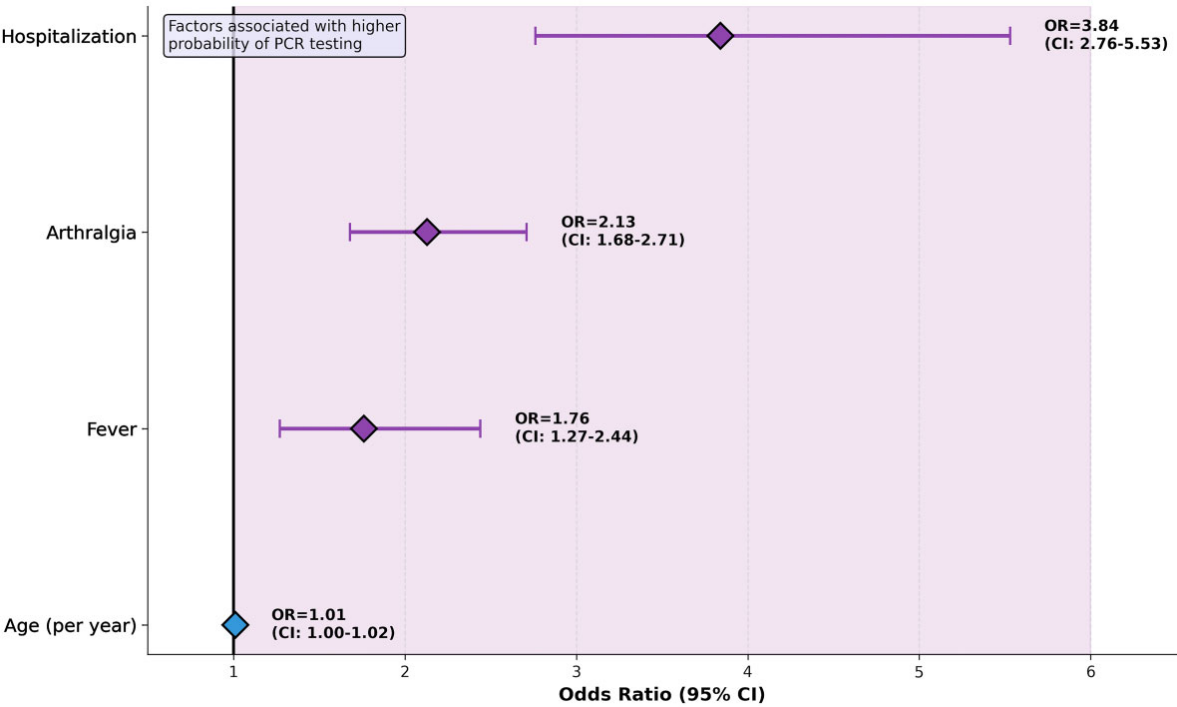
Selection by bias for RT-PCR testing in SINAN/DATASUS (Brazilian National Notifiable Diseases Information System). Statistical analysis: logistic regression for RT-PCR testing propensity; odds ratios (OR) with 95% confidence intervals (CI). Inverse probability weighting was used to adjust for selection bias.

Analysis of RT-PCR testing propensity in the SINAN/DATASUS dataset demonstrated significant selection bias (Figure 7). Hospitalized patients (OR=3.84; 95%CI: 2.76-5.53), as well as those presenting arthralgia (OR=2.13; 95%CI: 1.68-2.71) or fever (OR=1.76; 95%CI: 1.27-2.44), were more likely to undergo molecular testing. Age also influenced the probability of testing (OR=1.01 per additional year). Adjustment using IPW attenuated, but did not eliminate, differences in arthralgia prevalence between RT-PCR-positive cases and the population notified in the SINAN/DATASUS dataset.

### Temporal analysis and sequelae

Temporal analysis of the SINAN/DATASUS data for 2023 identified an epidemic peak between epidemiological weeks 12 and 20 (March to May), consistent with the expected seasonal pattern of arboviral infections in the region. Among the evaluated cases, 8.0% reported sequelae, with persistent arthralgia being the most frequent (75.0%), followed by chronic pain and residual edema.

## Discussion

This analysis, which compared RT-PCR-confirmed chikungunya cases with surveillance data from SINAN/DATASUS in Foz do Iguaçu, identified substantial discrepancies and critical challenges in clinical diagnosis and epidemiological surveillance. The results revealed distinct clinical-epidemiological profiles linked to the diagnostic confirmation method, indicating the presence of multiple biases in both medical practice and routine notification processes.

A notable observation was the high rate of initial clinical diagnostic errors, even among cases later confirmed by molecular testing (RT-PCR). This finding underscores the persistent challenge of differentiating chikungunya from other arboviral infections, particularly dengue, during early disease stages when clinical presentations often overlap. While this issue has been widely discussed in the literature, the present study provides quantitative evidence within a real-world surveillance and clinical care context. Additionally, the results indicated that current diagnostic strategies may rely excessively on traditional clinical patterns, lacking sufficient adaptation to the phenotypic variability revealed by molecular confirmation.

Symptom analysis emphasized the diagnostic importance of arthralgia, a cardinal symptom of chikungunya (6,35), which was more prevalent in the RT-PCR-positive group. However, comparison with SINAN/DATASUS data revealed a notable contrast: arthralgia prevalence was lower among laboratory-confirmed cases in SINAN/DATASUS, where late serological confirmation is common, and higher among cases confirmed by clinical-epidemiological criteria. This latter subgroup exhibited significantly greater frequencies of other symptoms, including myalgia, headache, nausea, vomiting, rash, arthritis, and retro-orbital pain, compared to the RT-PCR-positive group.

The findings indicated a symptom-based selection bias in the application of SINAN clinical-epidemiological criteria. Clinical classification appeared to be influenced by the presence of multiple symptoms, particularly those historically emphasized, such as arthritis and retro-orbital pain, even when these manifestations were rare or absent in molecularly confirmed cases. This discrepancy highlights a mismatch between the classical presentation perceived by healthcare professionals and the actual presentation observed in RT-PCR-positive cases. The complete absence of arthritis and the low frequency of retro-orbital pain in the RT-PCR-positive group contrast sharply with their prominence in clinical descriptions and diagnostic criteria (15), suggesting that molecularly confirmed presentations may diverge from prevailing clinical perceptions.

Hospitalization rates revealed a significant divergence. Cases confirmed by laboratory criteria in SINAN/DATASUS exhibited hospitalization rates more than twice those observed in the RT-PCR positive group (12.4 *vs* 5.5%), while clinically confirmed cases had markedly lower rates (1.3%). Analysis of testing propensity and multivariable models showed that hospitalized patients and those with arthralgia were more likely to undergo laboratory testing (PCR or serology). These results demonstrated a severity-related selection bias: surveillance systems relying on laboratory confirmation tended to overrepresent severe cases, whereas clinical confirmation included milder presentations. The RT-PCR-positive group, based on medical records, displayed intermediate hospitalization rates, and identified risk factors such as age over 60 years and autoimmune disease, which is aligned with existing literature (8,12).

These findings have significant implications for epidemiological surveillance. Clinical-epidemiological criteria require refinement, as the current overemphasis on arthritis and retro-orbital pain in SINAN/DATASUS, along with the negative association of symptoms such as myalgia and a positive tourniquet test with accurate diagnosis, suggests that existing criteria may result in misclassification and frequent confusion between chikungunya and dengue (15). The high rate of initial clinical diagnostic errors further supports the prevalence of this confusion in routine clinical practice.

Severity-related selection bias in laboratory testing can inflate morbidity estimates, which in turn affects epidemiological interpretation and influences public health decision-making.

Clinically, the findings underscore the importance of recognizing chikungunya's phenotypic heterogeneity. Oligosymptomatic profiles and atypical presentations, such as cases without fever or arthralgia and those with predominant rash or vomiting, are frequent (36) and may be underdiagnosed when only classical disease patterns are considered. Arthralgia demonstrated a high positive predictive value for PCR confirmation, reaffirming its significance as a priority symptom for clinicians. Additionally, identifying risk factors for hospitalization, including age over 60 years and autoimmune disease, facilitates improved risk stratification and targeted clinical management to optimize care and prevent adverse outcomes.

The comparison between RT-PCR-confirmed cases and SINAN/DATASUS surveillance data revealed substantial discrepancies in symptomatic profiles, hospitalization rates, and initial clinical diagnostic errors, including among cases later confirmed by PCR. Therefore, integration of diagnostic methods, epidemiological surveillance, and advanced statistical analysis is essential for a comprehensive understanding of the clinical and epidemiological behavior of chikungunya.

In conclusion, the identification of distinct symptomatic patterns and evidence of underdiagnosis in atypical presentations highlights the need for continuous training policies and regular updates to clinical protocols to reduce diagnostic inconsistencies. The findings also emphasize the importance of improving laboratory testing quality and adopting analytical models that correct biases, thereby enabling more accurate estimates to inform public health policies.

### Relevance and limitations

Considering the findings presented, this study is relevant for demonstrating, within a real-world clinical care and surveillance setting, that the method of diagnostic confirmation is associated with systematic differences in the clinical-epidemiological profile, revealing biases that may alter the interpretation of morbidity and severity indicators. Furthermore, the study supports improvements in both clinical risk stratification and the design of diagnostic and surveillance protocols that are more sensitive to phenotypic heterogeneity, including oligosymptomatic presentations.

The study is limited by its retrospective design, potential inconsistencies in medical records and SINAN/DATASUS notification forms, the lack of comorbidity information in the RT-PCR-positive dataset, and the inability to link individual cases between the two databases. Additionally, the analysis is confined to a single locality and time period, which may restrict the generalizability of the findings.

## Data Availability

The entire dataset supporting the results of this study was made available in a Data Repository: https://github.com/WelCode99/ArbovirosesDATA.git.
